# Successional Development of the Phototrophic Community in Biological Soil Crusts on Coastal and Inland Dunes

**DOI:** 10.3390/biology12010058

**Published:** 2022-12-29

**Authors:** Sandra Kammann, Ulf Schiefelbein, Christian Dolnik, Tatiana Mikhailyuk, Eduard Demchenko, Ulf Karsten, Karin Glaser

**Affiliations:** 1 Institute for Biological Sciences, Applied Ecology and Phycology, Rostock University, 18059 Rostock, Germany; 2 Botanical Garden, University of Rostock, 18055 Rostock, Germany; 3 Institute for Natural Resource Conservation, Landscape Ecology, Kiel University, 24098 Kiel, Germany; 4 M. G. Kholodny Institute of Botany, National Academy of Sciences of Ukraine, UA-01601 Kyiv, Ukraine

**Keywords:** algae, biocrusts, coastal dunes, lichen, moss, nutrient content, phototroph community composition, successional stages

## Abstract

**Simple Summary:**

This publication focuses on a comparative biodiversity survey of cryptogamic covers along a dune chronosequence in the Baltic Sea compared to an inland dune in northern Germany. Following these gradients, the cryptogamic vegetation accumulated more and more biomass and nutrients (carbon, nitrogen, and phosphorus). Further, a habitat-specific phototrophic community composition could be revealed. The enrichment of the biotic and abiotic components suggests cryptogamic covers as key players in geochemical processes, supporting sediment moisture and stability. Moreover, it highlights these communities as ‘ecosystem engineers’ playing a valuable part in nature protection e.g., preventing sediment erosion in coastal dune areas.

**Abstract:**

(1) Biological soil crusts (biocrusts) are microecosystems consisting of prokaryotic and eukaryotic microorganisms growing on the topsoil. This study aims to characterize changes in the community structure of biocrust phototrophic organisms along a dune chronosequence in the Baltic Sea compared to an inland dune in northern Germany. (2) A vegetation survey followed by species determination and sediment analyses were conducted. (3) The results highlight a varying phototrophic community composition within the biocrusts regarding the different successional stages of the dunes. At both study sites, a shift from algae-dominated to lichen- and moss-dominated biocrusts in later successional dune types was observed. The algae community of both study sites shared 50% of the identified species while the moss and lichen community shared less than 15%. This indicates a more generalized occurrence of the algal taxa along both chronosequences. The mosses and lichens showed a habitat-specific species community. Moreover, an increase in the organic matter and moisture content with advanced biocrust development was detected. The enrichment of carbon, nitrogen, and phosphorus in the different biocrust types showed a similar relationship. (4) This relation can be explained by biomass growth and potential nutrient mobilization by the microorganisms. Hence, the observed biocrust development potentially enhanced soil formation and contributed to nutrient accumulation.

## 1. Introduction

Coastal dunes as a transitional zone between the ocean shore and the terrestrial environment are shaped by harsh climatic and environmental conditions. These include strong seasonal fluctuations in temperatures near the sediment surface, drought, and wind, the latter causing constant sand movement, thereby shaping dunes [1]. Sandy dune soils are characterized by low soil organic matter content along with low nutrient concentrations [2]. Moreover, flooding events and salt spray have a high impact on substrate mobility [1,3]. Therefore, dune formations undergo a continuous succession driven by wind, waves, and storm events, as well as acidification due to precipitation (acid rain), shaping the dune geomorphology. These effects are important close to the sea, while more inland biomass typically accumulates creating a set of heterogeneous dune subsites [1]. The dune areas shaped by these mechanisms represent chronosequences, a geomorphological sequence of dune successions. In the ideal case, chronosequences differ only in the time since the onset of their formation, but all other abiotic factors during pedogenesis remained relatively steady [4]. Therefore, chronosequences are used for studying temporal habitat dynamics, reflecting vegetation and soil development [5]. A dune chronosequence starts with small foredune formations close to the shore, followed by mobile yellow dunes, which are still highly influenced by flooding and storms. Grey dunes connect the yellow dunes with the mature dunes, which are often forested [6]. Such a process takes place over a relatively long time scale (100–1000 years [7]). The effects of extrinsic stressors and intrinsic mechanisms (e.g., succession) can drive a dune ecosystem toward a particular assemblage of microbial and vascular plant communities and lead to different dune types characterized by unique geomorphology and vegetation patterns [8]. The establishment of a vascular plant cover is challenging due to the mentioned stressors. That, in turn, facilitates the colonization by stress-tolerant microbial communities, such as cryptogamic ground covers.

Cryptogamic ground covers are characteristic growth forms on top of various terrestrial soil or rock surfaces [9]. These living covers are of diverse appearance and include biological soil crusts (biocrusts). Biocrusts are particle-associated communities shaped by autotrophic and heterotrophic microorganisms, lichens, mosses, and their by-products, forming a coherent layer on the soil surface’s uppermost millimeters [10]. Due to the wide range of ecosystem functions, biocrusts affect the inhabiting ecosystem processes and can be closely linked to soil functionality [11,12]. As pioneer microbial communities, they support the initial habitat colonialization by producing organic matter among others [13]. Several studies focused on different mechanisms by which biocrusts can increase soil stability [14,15], which are important when considering soil erosion and blowouts [16]. Fischer et al. [17] refer to the advantage of thicker moss-dominated biocrusts regarding their water-holding capacity compared to thinner biocrusts. A study by Gypser et al. [18] proved this trend. Further, a high water holding capacity has been shown to reduce infiltration though this will depend on soil type, ecological context, and climatic region [11,19].

Phototrophic biocrust organisms are important contributors to the global photosynthetic carbon assimilation within the community biomass [20]. Moreover, biocrust cyanobacteria contribute to global terrestrial biological nitrogen fixation [9,21]. Former studies could also prove the importance of biocrusts in nutrient cycling [22], such as phosphorus [23,24,25,26]. The performance of all these collectively mentioned ecosystem functions is strongly related to the dominating biocrust-forming community and the successional stage of the respective biocrust cover [27,28]. A successional development can be seen as a sequence of species colonizing an open space. Thereby, prominent mechanisms interlock, namely facilitation, tolerance, and inhabitation [29]. In addition to species that depend on mechanisms, e.g., the dependency upon earlier species; biocrust succession depends on many environmental factors, namely temperature [30], radiation, topography, soil texture [31,32], microclimate [33], water availability [17,31], and nutrient availability, as well as the effect of seasons [34]. All those factors can either support biocrust development from early to mature thick moss and lichen-dominated communities or limit the further succession of biocrust covers, retaining them in early successional stages. Along with the changing environmental and abiotic variables, and geographical regions, the dominant organisms of biocrusts change and thereby, in turn, biocrust ecosystem functions, too. Biocrust successional development is, therefore, a process over time and in space influenced by multiple factors resulting in a heterogeneity of the community structures [35]. The time frame for biocrust succession was extensively investigated in various studies leading to an obvious outcome [36]. A current review by Kidron et al. [37] critically analyzed available estimates of the biocrust recovery time. The presented systematic measurements and theoretical considerations approve a relatively short-term recovery of biocrusts (cyanobacterial biocrusts: 5–10 years, lichen- and moss-dominated biocrusts: 10–20 years). The various stages of biocrust succession, caused by the changes in the species composition, allow the establishment of an abstracted categorization system for biocrusts. A study conducted by Büdel et al. [38] distinguished between seven main types of biocrust types along a > 2000 km long transect in Africa which were further categorized into three successional biocrust stages: early, intermediate, and late successional stages. A similar classification system, even though in smaller steps, was published by Williams et al. [39]. In the latter publication, biocrust types were classified by the functional phototrophic groups dominating the biocrust cover and the respective ecosystem functions (N-fixation, primary production, or soil stabilization).

Most studies focused on biocrust community development, functions, and changes in arid or semi-arid regions [38,40,41]. Much less is known for biocrusts in coastal sand dunes of temperate regions. Fernández-Alonso et al. [42] provided insight into the effects of increasing aridity and biotic cover on soil attributes and functions in coastal dune ecosystems of the Iberian Peninsula. Likewise, Schulz et al. [43] conducted a pioneer biodiversity survey of biocrusts in sand dunes in the Baltic Sea coast. This study highlighted the ecological importance of biocrusts in influencing soil properties, such as increasing total phosphorus concentrations, and their high site-specific diversity of microorganisms. However, the specific dune developmental stage was neglected here. In addition, Schaub et al. [44] focused exclusively on early successional biocrust stages in yellow dunes in the Baltic Sea. Still, information on community changes across biocrust successional stages along the natural dune development is missing, although they can be assumed to affect the ecosystem functionality, nutrient cycling, and further vegetation colonization of coastal dunes significantly. 

Therefore, in the present study for the first time, shifts in species composition, biocrust coverage, and biomass accumulation along dune successional gradients were investigated. The biocrust developmental patterns were linked with the dune successional stage along a dune chronosequence in the Baltic Sea compared to that of an inland dune in northern Germany. In order to uncover a link between the biogeochemical cycles of N, C, and P and the biocrust biodiversity correlations between species richness and nutrient status were investigated. It was assumed that (I) the phototrophic biocrust community composition shifts from a thin unstable topsoil layer of algae to stable moss and lichen-dominated ‘mature’ communities along each dune chronosequence. (II) The locational differences between the coastal and inland dunes will be reflected in the site-specific species composition of the respective biocrusts. A further aim was to characterize and compare the biocrust and sediment characteristics and nutrient contents of the different biocrust types between the sampling sites and along the dune successional stages.

## 2. Materials and Methods

### 2.1. Study Sites

**Figure 1:**
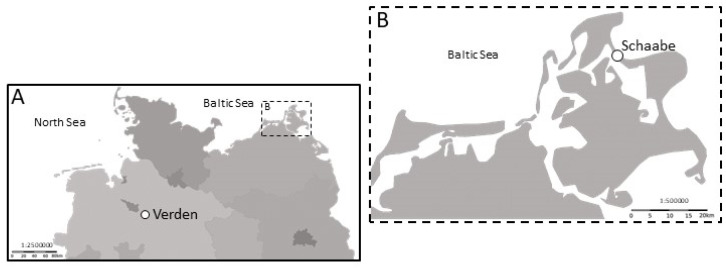
The inland dune in Verden and the island of Rügen in the northern part of Germany (**A**). A close-up of Rügen island (**B**) and the investigated dune sampling site in the Schaabe spit (circle).

Biological soil crust samples were collected in April and May 2020, along two dune succession sequences in northern Germany (Figure 1). Samples from an inland dune area (52.93865, 9.24917) in Verden (Aller, Lower Saxony, Figure 1A) were taken. This area is located within the nature reserve ‘Dünengebiet und Halsetal bei Verden-Neumühlen’ and includes a fauna–flora habitat area (nature protection area network Natura 2000). The second contrasting sampling site was located in the Schaabe (54.60318, 13.38872), a coastal dune spit in the Baltic Sea island of Rügen (Mecklenburg–Western Pomerania, Figure 1B). This spit connects the peninsulas of Jasmund and Wittow. The dune chronosequence under investigation belongs to the landscape conservation area ‘Ostrügen’. 

Both dune complexes are disturbed by anthropogenic impacts through tourism in the past. Trampling the dunes is prohibited but cannot be excluded. Moreover, even environmental protection measures, such as an adjacent reforestation area to curb further dune movement, might impact the dune development. In May 2020, the German Meteorological Service recorded a monthly precipitation of 14 mm total (an annual total of 543.3 mm) and a monthly average temperature of 10.7 °C at the ‘Arkona’ measuring station, which is close to the Schaabe sampling site. In April 2020, the monthly mean temperature for the inland dune in Verden was 9.9 degrees (measured at the Rotenburg station (Wümme); the nearest measuring point was ~24 km away). The monthly precipitation sum, however, could be determined near the inland dune, in Verden–Dauelsen, of 9.7 mm (an annual total of 651.4 mm). Meteorological data is from the DWD Climate Data Center (CDC), The monthly mean of the station measurements of the air temperature at a 2 m altitude in °C for Germany, version v21.3, retrieved on 16 November 2022, and the monthly sum of the station’s measurements of precipitation depth in mm for Germany, version v21.3, retrieved on 16 November 2022.

### 2.2. Sampling

#### 2.2.1. Transects

**Figure 2:**
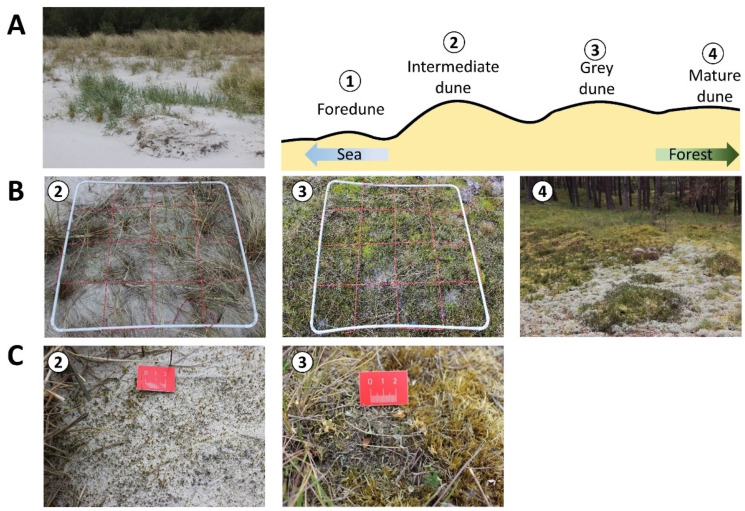
An overview of the transect in the Schaabe spit (Rügen) (**A**). Numbers indicate the sampling plots. Foredune (FD) = 1, intermediate dune (ID) = 2, grey dune (GD) = 3, and mature dune (MD) = 4. Close-ups of the biocrust-holding sampling plots (**B**) and detailed pictures of the dominant biocrust types in the respective plot (**C**). The white PVC frame marks each sampling plot. The red rope within this frame divides it into 16 subplots (0.0625 m^2^).

**Figure 3:**
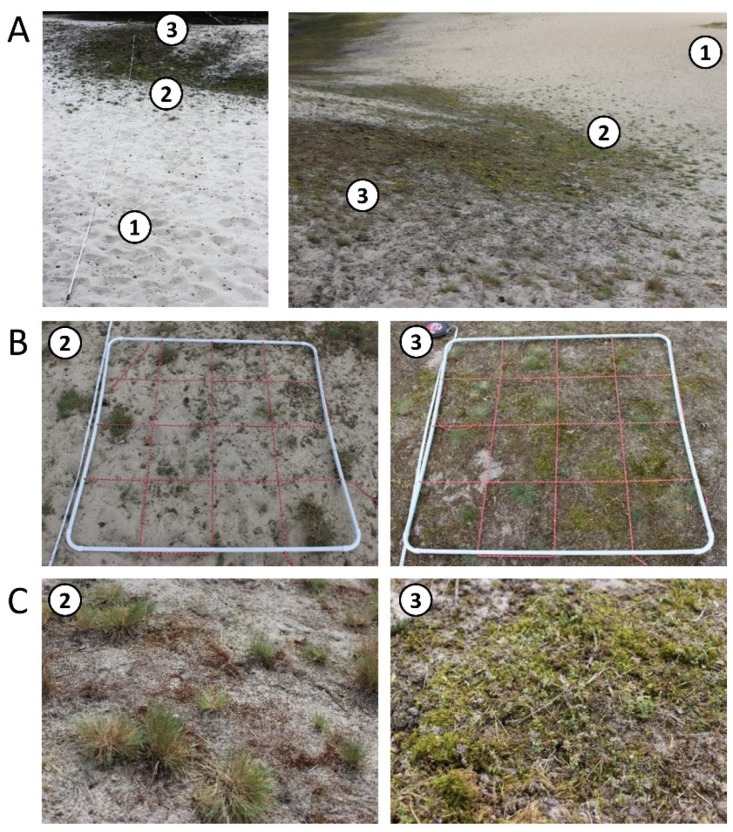
An overview of the transect in the inland dune in Verden (Aller); numbers indicate the sampling plots (**A**). Dune center (DC) = 1, dune slope (DS) = 2, and dune forest (DF) = 3. Close-ups of the biocrust-holding sampling plots (**B**) and detailed pictures of the dominant biocrust types in the respective plot (**C**). The white PVC frame marks each sampling plot. The red rope within this frame divides it into 16 subplots (0.0625 m^2^).

Biocrust samples were taken along one transect per study site. Each transect was established without prior consideration of the existing vegetation. The main focus of the transect was its orientation through a natural dune chronosequence that covered the different dune types. The transect in the Schaabe spit started from the shoreline to the first dune formation identified as a small foredune (FD) and passed through a former grey dune which gets continuously covered with a large amount of sand due to wind transport during storm events. Hence, the surface area of this dune is covered with several centimeters of mobile sediment. In this study, it will be considered as an interface between a fixed grey dune being frequently repulsed to a more mobile yellow dune phase. This hereinafter is called the intermediate dune (ID) stage and is an abstract recreation, and not a scientifically established term. This transitional dune stage is exemplary of the dynamic processes constantly shaping the dune morphology. The intermediate dune was followed by a grey dune area (GD). With decreasing sand accumulation, increasing sand stabilization, and vegetation cover, supporting organic matter accumulation, this dune type can develop. The transect ended in the mature dune area (MD), which was already overgrown with pine trees. The total length of this transect was 31 m. The transect in the inland dune in Verden started in the center of the dune area (DC) characterized by mobile bare sand representing a typical deflation hole. It crossed a dune slope (DS) where changes in vegetation cover were obvious, finally reaching the crest line of the parabolic dune. Close to the dune crest, the transect ended in a mixed dune forest area (DF) dominated by pine trees. The total length of this transect was 34 m. Along each transect, these described different successional dune stages were selected and further named dune subsites. At each subsite, a plot of 1 m^2^ was established and used for further vegetation analyses, biocrust, and sediment sampling. These plots were randomly placed within each described dune type and delimited by a 1 × 1 PVC frame. The plots covered a representative area of the present biocrust community. Each of the sampling plots was further divided into 16 equal subplots (0.0625 m^2^) by a grid made out of red rope stretched within the PVC frame (compare Figure 2 and Figure 3). 

In total, seven sampling plots at two study sites were under investigation in this study. Along the Schaabe spit transect, four subsites with one sampling plot each were established (Figure 2).

Three subsites were established in the inland dune in Verden (Figure 3). Altogether, five out of these seven plots showed biocrust growth in different successional stages.

#### 2.2.2. Vegetation

In preparation for the vegetation survey, seven different functional groups were defined describing the overall surface coverage (Table S1). These included four biocrust functional groups based on the established categorization systems by Büdel et al. [38], Lan et al. [32], and Williams et al. [39] but were slightly modified. Thin (1–3 mm) green algae-dominated biocrusts (GA) were defined as early successional stages. Later successional stages, in which the green algal biocrusts became slightly thicker (3–8 mm) and moss-covered, were defined as the intermediate successional biocrust stage (GA/M). Mossdominated biocrusts (MD) and those that were additionally lichenized (MD/L) characterized the mature successional stages of biocrusts. This study assumes the pre-presence of early algal biocrust before the later establishment of mature moss and lichen biocrusts and thus refers to the research results of Lan et al. [32], Williams et al. [39], and Langhans et al. [45]. Vascular plants and litter (dead material, e.g., pine needles, leaves, and branches) were two of the non-cryptogamic but still biotic functional groups. Bare sediment was the only abiotic functional group. 

The predefined functional groups were recorded within each plot according to the point intercept method by Levy and Madden [46]. A 25 cm × 25 cm (0.0625 m^2^) grid of 25 intersections was placed randomly into four of the sixteen subplots per quadrat (Figure S1). Within each subplot, the functional groups were recorded by 25-point measurements according to the approach of Williams et al. [39]. Therefore, a metal pin was dropped next to each intersection and the ground covering the functional group, including bare sediment, was recorded. That allowed 100 point measurements per sampling plot (1 m^2^).

#### 2.2.3. Sample Collection

Petri dishes (92 mm in diameter) were used for biocrust sampling. Each Petri dish was dropped and pushed gently into the respective biocrust. A spatula was then used to lift and separate the biocrust from the underlying sediment. The biocrust distribution in each sampling plot (1 m^2^) showed a high heterogeneity concerning the respective functional groups. The established 16 subplot grids from the vegetation survey were used again to collect the samples randomly. The grids were arranged in rows labeled with letters (A–D) and columns labeled with numbers (1–4). A random generator was used to decide from which of the 16 subplots biocrusts should be taken. One Petri dish sample was collected from one randomly selected subplot per plot.

Within each plot (1 m^2^), nine biocrust samples using Petri dishes were collected in total (one per subplot). Three of them were dedicated to chlorophyll *a* analyses, as well as for algae community cultivation, direct microscopy, and identification (three replicates per sampling plot from each study site in total). Additionally, six biocrust samples were taken for analyses of biocrust and sediment characteristics (water content and organic matter content, and nutrient concentration). The three biocrust samples for chlorophyll *a* analyses and the isolation of cultivable algae were wrapped with aluminum foil and stored in a cooling box for transportation. One additional sediment sample was collected in unvegetated areas within each of the seven sampling plots and stored in zip-lock bags. These samples represented the biocrust-free area of each plot and were used for sediment pH measurements. All mosses and lichens detected in the sampling plots were collected by hand and stored in paper bags. In the central plot (DC) in the inland dune and in the foredune (FD) in the Schaabe spit, no biocrusts were found. For keeping the sampling design at each plot uniform, samples were also taken from these two plots using Petri dishes even when no biocrusts were visible.

#### 2.2.4. Sample Preparation and Analyses of Biocrust and Sediment Characteristics

For chlorophyll analyses, three subsamples were punched out of each Petri dish using a cork drill (Ø 1.5 cm) to ensure they represented the same area for all samples. Those subsamples were treated as replicates per Petri dish and stored separately in a 15 mL falcon tube and frozen at −20 °C until chlorophyll *a* extraction. Hence, nine chlorophyll *a* measurements represented one sampling plot. This resulted in 63 measurements in total for this study (7 sampling plots × 3 petri dishes (replicates) × 3 subsamples per dish). The remaining biocrust or sediment in each Petri dish was air-dried for further algae isolation and identification and stored in the dark.

In the lab, biocrust and sediment samples used for further analyses were removed from the remaining six Petri dishes per plot. A razor blade was used to separate the visible biocrust or uppermost sediment layer from the underlying sediment. Three of them were used for biocrust characteristically (water and organic matter content) and the remaining three for nutrient (carbon = C_t_, nitrogen = N_t_, and phosphorus = P_t_) analyses. For biocrust characteristically analyses, the biocrust material from each Petri dish was weighed for fresh mass (FM) determination. Afterward, the samples were dried at 105 °C for 24 h and weighed again to determine the dry mass (DW) and calculate the water content. The organic matter (OM) content was calculated based on the weight loss after combustion at 450 °C for 5 h. The moisture content was expressed as a percentage of total fresh mass (% FM) and the organic matter content as a percentage of total dry mass (% DW). Each of the samples for nutrient analyses was further sieved (a 2 mm mesh size). From the now homogenized samples, three subsamples each were filled into PVC tubes for further analysis. This resulted again in 63 measurements in total for this study (7 sampling plots × 3 petri dishes (replicates) × 3 subsamples per dish). As a sediment characteristic, the pH of each of the crust-free sediment samples was measured in calcium chloride (0.01 M) solution after one hour (*w/v* ratio 1:4) with a pH meter (METTLER TOLEDO SevenMulti, Gießen, Germany).

### 2.3. Analyzed Parameters

#### 2.3.1. Nutrient Analyses

Total carbon and nitrogen (C_t_ and N_t_) were determined by dry combustion using the elemental analyzer (UNICUBE^®^ Elementar Analysensysteme GmbH, Langenselbold, Germany). For measurements of algae-dominated biocrusts containing a high amount of associated sand grains, 100 mg was used. For thicker, biomass-rich biocrusts, 50 mg per sample was sufficient. Total phosphorus (P_t_) was extracted from 500 mg of air-dried material by microwave-assisted digestion using aqua regia solution (3:1 hydrochloric acid—nitric acid). The concentration in the extract was measured by inductively coupled plasma optical emission spectroscopy at a 214 mm wavelength (ICP-OES Optima 8300, PerkinElmer, Inc. 710 Bridgeport Avenue Shelton, CT 06484-4794, USA).

#### 2.3.2. Chlorophyll a

Chlorophyll *a* (Chl *a*) content was used as a proxy for the photosynthetic biomass (chlorophyll *a* mg m^−2^) in biocrust (biocrust-holding plots) and sediment samples (plot DC from Verden and FD from Schaabe). The samples were each extracted in a 15 mL falcon tube. A total of 0.1 g of MgCO3 was added to each sample to avoid acidification. Chlorophyll *a* was extracted in 3 mL of 96% ethanol (*v/v*) for 30 min at 78 °C. Samples were shaken afterward and cooled on ice for 10 min followed by centrifugation at 5088 *g* for 5 min at 5 °C to decrease turbidity. The supernatant was carefully pipetted into a 1 cm quartz cuvette. A spectrophotometer (Shimadzu UV-2401 PC, Kyoto, Japan) was used for measuring the Chl *a* absorbance at wavelengths of 632, 649, 665, and 696 nm. The chlorophyll *a* content was calculated according to [47] and normalized to a square meter (m^2^).

Chl *a* (g m^−3^) = 0.0604 × (A_632nm_ − A_750nm_) − 4.5224 × (A_649nm_ − A_750nm_) + 13.2969 × (A_665nm_ − A_750nm_) − 1.7453 × (A_696nm_ − A_750nm_) (1)

#### 2.3.3. Algae Isolation, Community Cultivation, and Identification

Further processing of samples and identification of the most frequent and dominant species were conducted in the laboratory. To obtain enrichment cultures, fragments of biocrusts were placed in Petri dishes with Bold Basal (1N BBM) agarized medium [48]. Cultures were grown under standard laboratory conditions with a 12 h alteration of light and dark phases and irradiation of 25 μmol photons m^−2^ s^−1^ at a temperature of 20 ± 5 °C. Microscopic study of these raw cultures began in the third week of cultivation. Morphological examinations were performed using an Olympus BX53 light microscope with Nomarski DIC optics (Olympus Ltd., Hamburg, Germany). Micrographs were taken with a digital camera (Olympus LC30) attached to the microscope and processed by the Olympus software cellSens Entry. Direct microscopy of rewetted samples was performed in parallel with cultivation for evaluation of dominating species of algae and cyanobacteria in the original samples. Morphological identification of the biocrust organisms was based mainly on Ettl and Gärtner [49] for green microalgae, and on Komárek [50] for cyanobacteria, as well as on some monographs and papers devoted to taxonomic revisions of the taxa of interest [51].

#### 2.3.4. Moss and Lichen Identification

Moss and lichen samples were air-dried after collection. For determination, a microscope with a maximum magnification of 400× was used. Morphological identification of mosses followed Frahm and Frey [52] with taxonomical reference to Hodgetts et al. [53]. Lichens were determined according to Wirth et al. [54] and followed the nomenclature concept provided by Printzen et al. [55]. Morphologically critical species of the genus *Cladonia* were additionally analyzed by thin-layer chromatography, according to Culberson and Ammann [56] in solvent system A. 

### 2.4. Statistical Analyses

All statistical analyses were conducted using R version 4.2.1 (R Core Team (2022) Vienna, Austria). The mean of the replicate samples per quadrat was calculated for further statistical analysis. Analysis of similarities (using the ‘anosim’ function in R) was performed to test for significant differences in the measured biocrust and sediment characteristics and nutrient contents between the sampling sites and across the transect plots in both dune areas. Data collected for the analysis of vegetation patterns, obtained via the point intercept method, were used to calculate the percentage of areal coverage by each functional group for each sampling plot and visualized as a stacked bar graph. Differences in the overall community composition of biocrusts from the two sampling sites, Verden and Schaabe, and along the transects following the dunes succession stage were visualized by non-metric multidimensional scaling (nMDS) using the vegan package and the Bray–Curtis dissimilarity index implemented in R. Correlation of community composition with biocrust and sediment plot characteristics and nutrient contents were statistically analyzed via permutational multivariate analysis of variance (PerMANOVA) (with the ‘adonis’ function in R) using the Bray–Curtis distance matrix implemented using the vegan R package [57]. Biocrust and sediment characteristics and nutrient parameters that significantly correlated with community composition were added to the nMDS plot. The person correlation (the ‘cor’ command in R) was further used to test the nutrient contents individually for a possible correlation with the species richness of each phototrophic group. The total species number of each phototroph group (algae, moss, and lichen) per sample was used as the measure of alpha diversity. As a measure of beta diversity, the presence/absence data of the biocrust-inhabiting species were visualized with a Venn diagram using Microsoft Excel 2013 (Microsoft Corporation, Redmond, WA, USA).

## 3. Results

### 3.1. Biocrust and Sediment Characteristics, and Nutrient Analyses

The pH of the sediment in the Schaabe spit conspicuously decreased starting from the foredune (6.39) to the mature dune (3.98) (Table 1). A similar trend was observed at the inland dune in Verden. A pH value of 4.49 was measured in the sediment of the dune center and decreased following the transect to the sampling plot closest to the dune surrounding forest (3.35) (Table 1). Hence, the pH values continuously declined from slightly to moderate acidic along both natural dune succession sequences. The organic matter content (OM) in the Schaabe spit increased along the transect when starting at the foredune (uppermost sediment layer*) with very low contents (0.11 ± 0.01% DW). OM accumulated toward the mature dune up to 25.19 ± 7.7% DW in the biocrust (Table 1). For the inland dune, the OM content showed a similar pattern. It started in the biocrust-free sediment of the dune center with an OM value < 1% DW and increased in the biocrusts from 3.3 ± 0.72% DW at the dune slope to 24.03 ± 2.66% DW at the plot close to the dune forest (Table 1). The gravimetric water content in the Schaabe spit ranged between 0.52 ± 0.83% DW (FD, uppermost sediment layer*) and 1.22 ± 0.56% DW (MD, biocrust) (Table 1). As for the pH and OM content, the water content increased along the dune succession gradient toward the mature dune. The same applied to the samples from the inland dune. 

The total carbon concentration (C_t_) along both transects increased following the dune successional gradient inland and reached a maximum of 99.63 ± 4.59 g kg^−1^ in the biocrusts of the dune area close to the forest at the inland dune. The same trend was observed for the total nitrogen (N_t_) concentration at both sampling sites (Table 2). Likewise, the total phosphorus (P_t_) concentration along both transects increased toward older dune successional stages. The lowest P_t_ concentrations comparing both sites were measured in the uppermost sediment layer of the dune center (29.89 ± 2.29 mg kg^−1^) and the biocrusts of the dune slope (71.85 ± 11.44 mg kg^−1^) of the inland dune. On the spit, the lowest P_t_ concentration was measured in the uppermost sediment layer of the small foredune (113.9 mg kg^−1^). Like in the inland dune, P_t_ concentrations increased toward the mature dune area. The highest P_t_ concentration at both sites was measured close to the forest (291.25 mg kg^−1^) in the biocrust of the inland dune (Table 2). No significant difference in the nutrient content between sites could be detected. However, along each dune chronosequence, the nutrient content differed significantly between the varying dune types (inland dune: *p* = 0.004 and coastal dune: *p* = 0.001, anosim).

### 3.2. Vegetation Surveys

**Figure 4:**
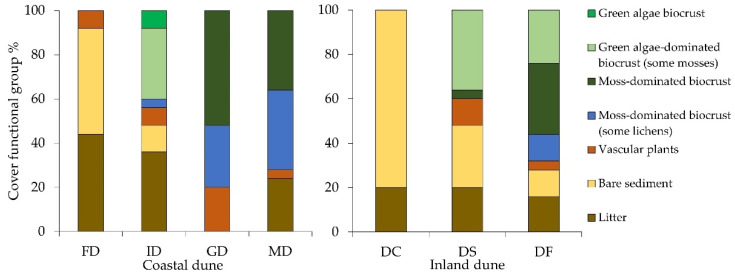
A summary of the vegetation survey. Percentage of the area covered by the different functional groups, as determined by the point intercept method along the two transects. Site: coastal dune (Schaabe). Dune types: FD = foredune, ID = intermediate dune, GD = grey dune, and MD = mature dune. Site: Inland dune (Verden). Sampling plots: DC = dune center, DS = dune slope, and DF = dune forest.

Biocrust cover increased along the dune succession gradient at both study sites. The percentage of coverage of each defined biocrust functional group reflected the vegetation dynamics along with the dune succession (Figure 4).

In the Schaabe spit, the first dune type under investigation was a small foredune. No cryptogamic cover was found within this dune type (Figure 4). The surface was mainly bare sediment (48%) or covered by organic litter (44%). Occasionally occurring vascular plants (8%) were *Ammophila arenaria* (L. LINK), which were planted for coastal protection management of dunes. Following the transect inland into the intermediate dune, the earliest biocrust covers were detected. These covers were dominated by green algae, defined as initial biocrusts, smaller mosses, and occasionally lichens growing only on plant litter. Here, biocrusts were the dominant surface cover with 44%, followed by the surface cover of a litter layer (36%) (Figure 4). The grey dune exhibited the highest biocrust coverage of all dune types (80%) and showed a change in dominant biocrust functional groups. Moss-dominated biocrusts (52%) replaced those dominated by green algae. Additionally, moss-dominated biocrusts associated with lichens covered 28% of the grey dune (Figure 4). In contrast to the intermediate dune, where lichens occurred only occasionally on plant litter, they became more dominant and area-covering in the grey dune. In the mature dune, a thick moss and lichen carpet, characteristic of more developed successional biocrust stages, dominated the dune surface. The functional groups of moss-dominated and moss-dominated biocrusts associated with lichens overgrew the dune in equal parts (both 36% areal coverage) (Figure 4). In addition, the litter layer was formed mainly by pine (*Pinus sylvestris* L.) needles and covered 24% of the surveyed dune surface. Bare sand was rare in the grey and the mature dune. Most likely human trampling, animals, or abiotic factors, such as wind and rain, caused such small bare sediment spots in these dune types.

The proportion of bare sediment decreased along the transect at the inland dune in Verden and was replaced by biocrusts in the oldest dune area close to the forest. Bare sediment (80% areal coverage) and organic litter (20%) characterized the center of the inland dune (Figure 4). The first biocrusts along the transect were recognized on the dune slope. This sampling plot was dominated by early green algae biocrusts associated with the occasional occurrence of small mosses (36%) (Figure 4). Moreover, a few moss-dominated biocrusts could be observed (4%). The dune area close to the forest had the highest biocrust coverage of all three investigated subsites (68%) (Figure 4). Additionally, the first lichen development was found in the area close to the forest (12%). However, mature moss-dominated biocrusts were the characteristic functional group within this dune area (32%) (Figure 4). 

The chlorophyll *a* content of the biocrusts along the transects at both study sites was associated with the transition in species composition and resulting changes in dominant functional groups. In the inland dune, the chlorophyll *a* concentration was lowest in the dune center (DC = 5.86 mg m^−2^) and rose to 112.07 ± 20 mg m^−2^ on the dune slope where the green algae-dominated biocrusts and some mosses were most present. In the area close to the forest chlorophyll, *a* content was highest (210.8 ± 109.01 mg m^−2^). A similar pattern could be observed along the transect in the Schaabe spit. At the beginning of the transect in the foredune, no chlorophyll was measurable in the bare sediment. The moss-dominated biocrusts in the mature dune showed the highest values (287.63 ± 49.07 mg m^−2^).

### 3.3. Algae and Cyanobacteria

Within five biocrust samples and one sediment sample, thirteen algae species (nine in the inland dune, twelve in the Schaabe spit), and one cyanobacterium species (the DC plot in the inland dune) were detected using culture-dependent methods followed by morphological identification (Table 3). The richness of species was expressed as the total species number identified per plot. Along both transects, algae were detected with species numbers ranging between one and eight per sampling plot. Along the inland dune transect, the dune slope showed the highest algal species richness (eight out of fourteen species). In the Schaabe spit, the intermediate dune plot was most rich in species (eight out of fourteen species).

**Figure 5:**
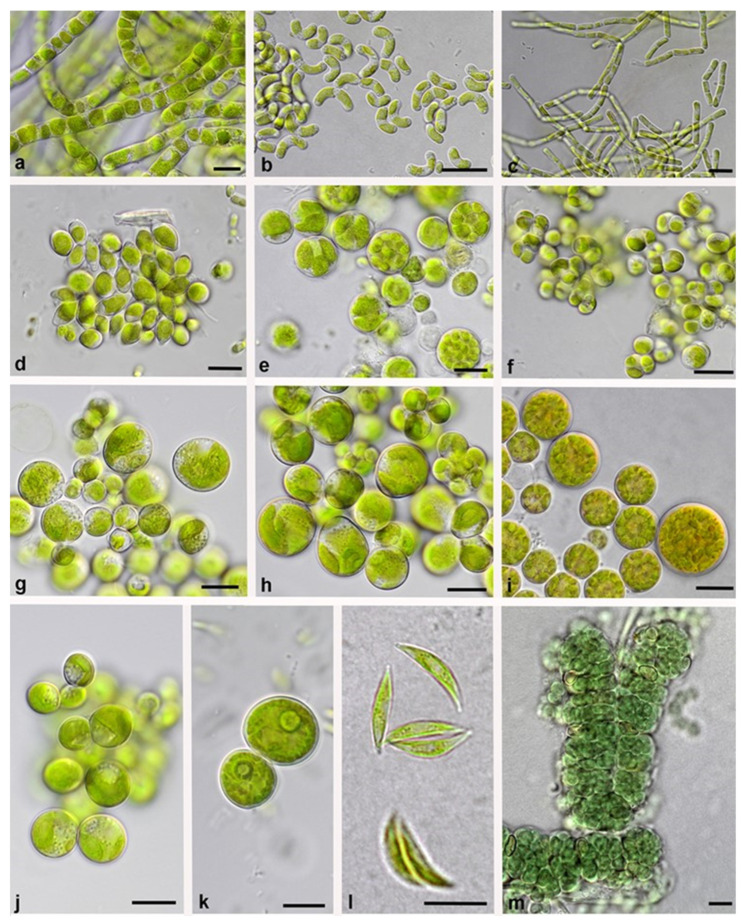
Representative algae and cyanobacteria from different dune succession stages along the two transects. (**a**) *Klebsormidium* cf. *subtile*, (**b**) *Stichococcus allas*, (**c**) *Stichococcus* cf. *bacillaris*, (**d**) *Chloroidium* sp., (**e**) *Watanabea* cf. *acidophila*, (**f**) *Diplosphaera chodatii*, *(***g**) *Parietochloris alveolaris*, (**h**) *Myrmecia* cf. *irregularis*, (**i**) *Bracteacoccus* sp., (**j**) *Elliptochloris subsphaerica*, (**k**) *Coelastrella* sp., (**l**) *Chlorolobion* sp., and (**m**) *Nostoc* cf. *edaphicum*. Scale bars are 10 µm.

Twelve species of Chlorophyta belonging to two classes were determined (three members of Chlorophyceae and nine members of Trebouxiophyceae). One species of Charophyta was detected and assigned to Klebsormidiophyceae. One species (*Nostoc* cf. *edaphicum*, Figure 5m) of Cyanobacteria was found. The most frequently detected taxa were *Myrmecia* cf. *irregularis* (Figure 5h) and *Stichococcus* cf. *bacillaris* (Figure 5c). Individuals of both genera were found at two sampling plots (*Myrmecia* cf. *irregularis*: DC and DS; *Stichococcus* cf. *bacillaris*: DS and DF) in Verden and three plots (ID, GD, and MD) in the Schaabe spit. Both species were followed by *Watanabea* cf. *acidophila* (Figure 5e) in four out of six samples. The other species occurred in half or fewer of the biocrust samples. *Nostoc* cf. *edaphicum* was detected once in the intermediate dune in the Schaabe spit. A total of 38.5% of all detected species were detected uniquely. Both study sites had 50% of the identified species in common (Figure S2A). Six of these shared species belonged to the Chlorophyta and one to the Charophyta.

### 3.4. Moss Species

Sixteen moss species were detected at five of seven sampling plots in this study. Across all five moss-holding plots, the species’ richness ranged between three and nine species. The grey dune plot in the Schaabe spit showed the highest species number of all investigated plots (nine out of sixteen species). The species’ richest plot along the inland dune transect was the one close to the forest (Table 4).

Fourteen species of Bryophyta were identified, mostly belonging to the class of Bryopsida and one species belonging to the class of Polytrichopsida. The remaining two species were assigned to the Jungermanniopsida in the phylum Marchantiophyta. The almost omnipresent species *Ceratodon purpureus* could be found at the inland dune sampling plots DS and DF, and ID, GD, and MD in the Schaabe spit. All other species occurred in two or fewer of the five moss-holding sampling plots. A total of 50% of all detected species were detected uniquely. The study sites showed only two out of sixteen identified species in common (12.5%), namely *Ceratodon purpureus* and *Dicranum scoparium* (Figure S2B).

### 3.5. Lichen Species

Twenty-seven lichen species were found at four of seven sampling plots in this study. (Table 5). Across all four lichen-holding plots, the species’ richness ranged between four and sixteen taxa. The intermediate and grey dune plots in the Schaabe spit had the highest species number of all investigated plots (16 out of 27 species). Along the inland dune transect, the sampling plot close to the forest was the only one on which lichens could be found. In this plot, four out of twenty-seven overall detected lichen species were observed (Table 5).

All determined lichen species belonged to families of the class of Lecanoromycetes, and 22 out of 27 species were members of the Lecanorales order. Four different orders were determined in total. *Cladonia furcata* was the only lichen species found in all lichen-holding plots in this study. *Cladonia furcata* and *Cladonia portentosa* were the only lichen species both study sites had in common (Figure S2C). Sand-inhabiting species dominated this survey (19 species). The other eight lichen species only grew on litter and plant biomass. Latter were exclusively found in the intermediate and grey dunes on the coastal dune. All litter layer-inhabiting species, aside from one (*Micarea misella*), settled primarily in subneutophil habitats. Following the coastal dune chronosequence toward the mature dune area, a continuous increase in acidophil lichen species occurred. A detailed table on the pH-characterizable location dependencies can be found in the supplementary material (Table S3).

### 3.6. Biocrust Community Composition

**Figure 6:**
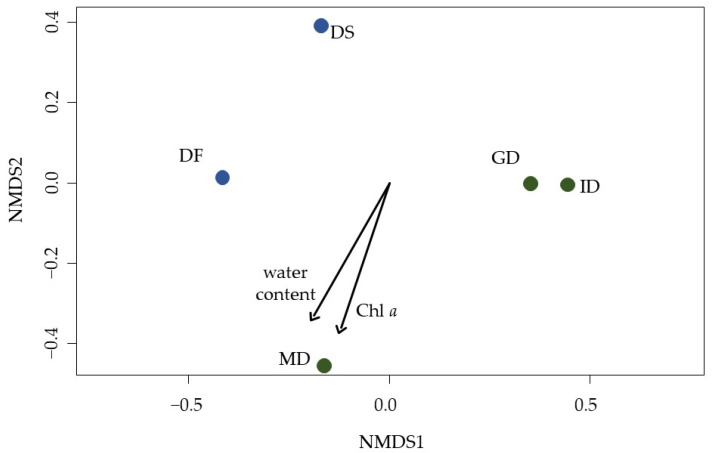
An nMDS plot visualizes the differences and similarities in the community composition of grouped lichen, mosses, and algae between the five sampling sites. Green: coastal dune (Schaabe). Dune types: ID = intermediate dune, GD = grey dune, and MD = mature dune. Blue: inland dune (Verden). Sampling plots: DS = dune slope and DF = dune forest. The black arrows indicate the influence direction of the two significantly correlated biocrust characteristics, namely water content and chlorophyll *a*. Stress = 0.

The community composition of moss, lichen, and algae in the biocrusts differed between the sampling sites and along the transects (Figure 6). Community composition in intermediate and grey dunes from Schaabe were similar to each other. The mature dune was quite dissimilar from the other two Schaabe plots and more similar to the latest stage in the transect of Verden. Both Verden plots differed from each other and the Schaabe sites. The earliest stage in the transect of the inland dune (DC) and the coastal dune (FD) had to be excluded from the analyses, as only two algal species and no moss or lichen species were detected. Statistical analyses using PerMANOVA verified that the community composition was significantly shaped by the sampling site (explained variance 42% and *p*-value < 0.02) and developmental stage of the dunes (explained variance 36% and *p*-value < 0.02).

The biocrust’s gravimetric water content (explained variance 39% and *p* = 0.07), and Chl *a* content (explained variance 33% and *p* = 0.08) tended to correlate with the change in the community composition (PerMANOVA) between the sampling sites and plots. The phototrophic species richness showed the trend to influence especially the total phosphorus content within the biocrust, even though this trend could not be statistically verified as significant. Looking at the phototrophic groups separately, the moss richness tends to be likely influencing the phosphorus content within the biocrust (Figure S3).

## 4. Discussion

### 4.1. Vegetation

Coastal foredunes and, equally, the center of the inland dunes, are characterized by strong winds resulting in a highly mobile substrate. Additionally, nearshore, higher sediment salinity and air-borne salt spray cause harsh conditions on coastal dunes. Along with a scarcity of nutrients, neither a stable plant nor a biocrust cover could be established at both study sites [1,3,6,58]. Consequently, the organic matter and chlorophyll *a* content were lowest on these mobile dune types. In addition, microorganisms, such as filamentous green algae or cyanobacteria, which might glue sand particles together by excreted exopolysaccharides [16,59,60], were missing. Hence, no coherent topsoil layer can be formed. Consequently, precipitation will easily infiltrate into loose sandy sediment [18]. The earliest successional stages of biocrusts were found in the chronologically following intermediate dune in the Schaabe spit and on the dune slope in the inland dune (Verden). They were mainly formed by green algae, covering approximately half of the dune’s surface, forming patchy and thin microbial phototrophic layers. The growth of mosses and lichens could be partially observed as well. However, the mature biocrust establishment was hardly observed in intermediate dunes due to the frequent disturbance by erosive wind forces leading to sand mobility [61] or due to water limitation caused by the low water-holding capacity of the sand [18,19]. Soil moisture can positively affect carbon and nitrogen fixation by microorganisms, as was observed for biocrusts in arid regions [36]. The accumulation of organic carbon by microbial biomass formation plays an essential role in early pedogenesis [62]. Organic carbon accumulation is controlled by the turnover rates of the soil’s organic matter [63]. The present organic matter accumulation was assumed to be based on faster biomass formation and litter input than decomposition within the studied two dune areas. It is assumed that the increase in the organic matter within the biocrust has a potential impact on the mechanisms of biogeochemical P cycling in the sediment. Inorganic bound P (e.g., Ca-phosphate minerals) can be solubilized from parent material either by the secretion of organic acids by microorganisms [64] or by an increase in the pH [65]. Phosphatases, produced by biocrust organisms, are known to hydrolyze organic phosphates releasing P (e.g., from dead cells) [66]. The increase in total P in the biocrusts along both chronosequences can mainly be explained by the increased biomass formation. Increased phototrophic biomass is revealed by the rising Chl *a* content. Depending on the sampling site, season, biocrust communities, and successional type, the Chl *a* content varies clearly. Lange [20] reported a Chl *a* content of up to 100 mg m^−2^ in biocrusts formed by cyanobacteria and eukaryotic algae, whereas lichen- or bryophyte-dominated biocrusts reached values above 900 mg m^−2^. Just as it is proposed by Büdel et al. [38], the presented study could prove a significant increase in the chlorophyll content in the biocrusts of the early successional stage to the later ones. In a semi-arid desert, Büdel et al. [38] measured a mean Chl *a* value of 118.9 ± 35.8 mg m^−2^ in an area with strong coverage of biocrusts with bryophytes. These findings fit in with the Chl *a* value of the dune slope of the inland dune, which was also dominated by green algae biocrusts associated with mosses (112.07 ± 20 mg m^−2^). The Chl *a* value of the intermediate dune (161.34 ± 56.51 mg m^−2^) and grey dune (150.92 ± 26.49 mg m^−2^) in the Schaabe spit exceeded this value slightly. One reason for these values could be their largely shared moss and lichen community composition. Along the dune chronosequences, the successional stages of biocrusts gain more biomass due to thicker green algae layers and their by-products. Moreover, the gain in biomass and sediment organic matter might be caused by the increased establishment of mosses. The Chla *a* values of moss-dominated biocrusts in the presented study (DF inland dune = 210.80 mg m^−2^ and MD coastal dune = 287.63 mg m^−2^) were significantly higher than those measured by Gypser et al. [35]. These authors reported a mean Chl *a* content of 68.9 mg m^−2^ of moss- and lichen-dominated biocrusts in an artificial temperate sand dune.

Moss-dominated biocrusts took over and covered about half of the investigated plot within the grey dune in the Schaabe spit. These biocrust types formed a denser and more coherent layer on the sediment surface without bare sediment. The growth of lichens increased significantly. The increase in moss and lichen biomass along the chronosequence characterized the transition from the intermediate dune to the established grey dune area [67]. The percentage coverage by the vascular plant was highest in this dune type, mostly dominated by Poaceae. The development of grey dunes was characterized by increased sand stabilization due to less sand accumulation [68]. Therefore, vegetation cover could expand and support the formation of an organic matter layer on the sediment surface [69]. Such a layer was composed of dead plant material along with living phototrophic and heterotrophic biomass, originating from the biocrust. A similar trend was reported by Gypser et al. [35], who showed a progressive increase in biomass and the total chlorophyll content from initial to moss- and lichen-dominated biocrusts at post-mining sites in Lower Lusatia, northeast Germany. The mature dune area landwards in the Schaabe spit and, respectively, the area close to the forest in the inland dune represented the oldest successional stages of dunes along the investigated dune chronosequences. They differed from (younger) dune types by a closed biocrust cover dominated by mosses with the highest occurrence of lichens. These cryptogamic covers grew under a light canopy of a coastal pine forest. Mature dunes, as the latest phase of dune succession, represented a high organic matter content as an indicator of early soil formation [70]. This assumption is consistent with the observations made in the present study and in line with Isermann [69]. Here, the organic matter accumulation originated from annual vascular plants, scrubs, and smaller trees. The observed increase in the biocrust water content could be ascribed to the thick layer of the phototrophic community dominated by mosses and lichens. Such mature biocrusts absorb water into the cellular mucilage using morphological adaptations, such as lamellae and filaments, and thus exhibit a higher water-holding capacity [11,71], thereby stimulating sediment water retention and increasing water availability [72], at least in the top layer. Chamizo et al. [19] additionally highlighted the later stages of biocrust succession, as dominated by lichens and mosses by their higher infiltration capability and water retention, finally leading to higher soil moisture. The presented results showed similar patterns and are in agreement with a study by Gypser et al. [18], in which green algae tended to reduce water infiltration due to the potential pore clogging by algal filaments and their usually high amounts of sticky extracellular polymeric substances (EPS) [16]. Further it was assumed that mosses in later successional biocrust stages probably reduce pore clogging and might have reduced the previous surface water runoff. In contrast, later successional stages of biocrusts, dominated by mosses and lichens, can absorb more water and reduce runoff compared to the algae-dominated biocrusts. Particularly, moss rhizoids can facilitate water infiltration into deeper soil layers [70].

The vegetation survey revealed a successional development of biocrust stages from a thin topsoil layer of algae to a stable moss and lichen-dominated ‘mature’ community along the two investigated dune chronosequences. An existence of early successional biocrust stages before the development to older stages can be assumed since algae species could still be detected in the older biocrust stages. That confirmed our first hypothesis. An increase in the proportion of the ground cover by biocrusts was detected along each transect. The oldest and most established dune types of each transect (DF = Verden and MD = Schaabe) showed the highest proportion of moss-dominated biocrusts compared with the other plots. These results are in line with a described strong zonation from grey to mature dunes regarding the vascular plant communities on the German Wadden Sea coast [69].

### 4.2. Species Composition Changes

In addition to the general shift in biocrust types (algae, moss, or moss- and lichen-dominated) along the chronosequences, a change in the species composition between the individual dune types was also evident. Only a few species were omnipresent along each chronosequence. Comparing both study sites, locational differences in the community structures (species presence/absence) between the coastal and inland dunes became obvious. While the algal community showed many similarities, the moss and lichen communities differed significantly between the two study sites. Based on these findings, the second hypothesis could be confirmed. the differing locational conditions of both sites were reflected in differing species composition of the biocrusts in sand dunes. Biotic (e.g., litter layer and plant association) and abiotic factors (e.g., storm events, flooding, and salt spray) differ typically for the respective study site. The resulting site conditions can be seen as causes leading to the local differences in the phototrophic species compositions of biocrusts.

#### 4.2.1. Algae and Cyanobacteria

Species belonging to the phylum Chlorophyta were mostly present at both dune chronosequences. High richness of Chlorophyta is characteristic of temperate habitats and forest soils [73]. Comparing the overall green algae and cyanobacteria species richness in the Schaabe spit with the latest studies on biocrust microbial community diversity in this area, Schulz et al. [43] indicated similarities but also conspicuous differences. Even though biocrusts are consistently formed by algae rather than cyanobacteria, species’ richness can differ. While Schulz et al. [43] detected 70 cyanobacterial and non-diatom algal taxa in association with biocrusts in coastal dunes in the Baltic Sea, the recent study could reveal only twelve. Since species composition in biocrusts very likely varies with season [34] this might be one reason for the dominance of green algae in the spring biocrusts and cyanobacteria in the autumn biocrusts (October—Schulz et al. [43] and April/May in this study). Schulz et al. [43] showed biocrusts consisting mainly of a few green algae and cyanobacteria in the early successional stages of dune development at their temperate study sites. This is in contrast with studies carried out in arid or semiarid deserts where cyanobacteria were always dominant in early successional biocrusts [12,31,38,74,75]. However, the presented findings are comparable to other studies conducted in temperate areas [76,77]. In temperate forests, Glaser et al. [78] recorded 52 algal species and only very few cyanobacteria in biocrusts. Additionally, 17 eukaryotic algae and 15 cyanobacteria species were found in initial biocrusts in a sand ecosystem in the northern upper Rhine valley (Germany) [45].

In the center of the inland dune, only a few algae taxa could be observed in the bare sand, even if they did not form a thin biocrust layer. Contrary to the foredune close to the Baltic Sea, microorganisms in inland dunes do not have to cope with salt and wind stress. This might facilitate their survival in the mobile sediment of the inland dune center. However, it cannot be excluded that the detected algal species were in dormant stages. These algae can act as pioneer colonizers of the mobile sand facilitating further stabilization and colonization. Light green algae-dominated biocrusts were the only ones containing cyanobacteria along the whole dune chronosequence. One reason for the rare occurrence of cyanobacteria could be the sediment conditions, in particular the pH. For optimum growth of cyanobacteria-containing biocrusts sediment, pH should be neutral to slightly alkaline [79]. Under acidic sediment conditions (pH less than 4 or 5), Brock [80] could confirm that cyanobacteria were completely absent. The sediment pH along the two dune chronosequences showed a landward strong decrease from 6.4 to 4.0 (coastal dune, Schaabe) and from 4.5 to 3.4 (inland dune, Verden). These acidic conditions in the inland dune might limit the growth of cyanobacteria [80]. The biocrusts in the intermediate dune in the Schaabe spit showed only slightly acidic conditions (6.1), and thus few cyanobacteria could grow. In addition to the geological impact on pH dynamics, the coniferous vegetation (e.g., *Pinus sylvestris* L.) typically leads to a decrease in the soil pH due to the microbial decomposition of pine tree litter [2]. Considering the shifts in vegetation cover along both dune chronosequences, the oldest successional dune areas were located under or close to the dune forest. These areas, dominated at both study sites by pine trees, could be one potential reason for the decrease in sediment pH and causing unfavorable growth conditions for cyanobacteria in the mature dune area. Nevertheless, it could be possible that not all cyanobacteria were detected due to chosen cultural approach. Previous studies showed the necessity to use different determination approaches e.g., morphological and molecular-based, revealing a more complete community structure of biocrusts [81,82].

The biocrusts on the dune slope of the inland dune mostly consisted of Chlorophyta and representatives of the Charophyta in the Klebsormidiophyceae class. Moreover, this dune’s successional stage was the species’ richest along the whole chronosequence. These findings were identical to those of the intermediate dune in the Schaabe spit. Here, most algal species could be detected. Representatives of the genus *Klebsormidium* were only found in this dune’s successional stage. Many studies point to *Klebsormidium* as a genus, holding species with a wide tolerance range in temperature, water availability, or sun radiation [83,84,85]. To cope with such extremely changing environmental conditions, Donner et al. [86] assume certain plasticity of the genus morphology as a benefit for adapting to these varying abiotic stresses. These filamentous algae are known as major biocrust-forming taxa [83]. Glaser et al. [78] could prove *Klebsormidium* was the most important biocrust-initiating alga in a temperate forest ecosystem. In an inland dune area in the Netherlands, Pluis [77] found *Klebsormidium* to be the initial green alga genus in the successional development of the biocrust community. Likewise, representatives of the genus *Klebsormidium* were found in dynamic dune successional stages along both transects, highlighting the initialization of biocrust development.

With further dune succession, the algal species richness declined. Four species of the family Trebouxiophyceae were found in the area close to the forest in the inland dune and the mature dune in the Schaabe spit, having two of those species in common. Such clear differences between the algal community structure of the initial and later successional biocrust stages were also shown in a sand ecosystem in the northern Rhine valley (Germany). Here, Langhans et al. [45] could show a decrease from 17 eukaryotic algae in early successional biocrust stages to 13 species in later successional biocrust stages, which is in common with this study.

#### 4.2.2. Mosses and Lichens

Moss- and lichen-dominated biocrusts were distinctive of older stages of development. In phytosociology [87], the development of a cryptogam layer marks the beginning of grey dune development, as drifting sand will not cover the small cryptogams. On the other hand, the cryptogam layer helps to fix a small amount of drifting sand and bring organic litter into the sediment.

Incipient moss and lichen growth could be described on the dune slope of the inland dune and the intermediate dune in the Schaabe spit, respectively. *Ceratodon purpureus* was found in both transitional stages of dune succession. This moss species is typical for mobile dune types due to its high tolerance to sand deposition. A study on the response of mosses to experimental burial by Martínez and Maun [88] could show a high tolerance of *C. purpureus* of up to a 7 cm thick sand layer. This species is the only one that the dune slope in the inland dune and the intermediate dune in the Schaabe spit had in common. Along both chronosequences, *C. purpureus* was observed in transitional dune types, as well as in later dune stages, such as the area close to the forest in the inland dune and on the fixed grey dune in the Schaabe spit. These findings are in line with Gypser et al. [35], who described the growth of *C. purpureus* as an initial moss, as well as dominant in mature moss and lichen biocrusts. Another species only found on the dune slope of the inland dune is *Syntrichia ruraliformis*. This species is highly desiccation-tolerant [89,90]. This trait is favorable for moss growing on dune types that are under constant abiotic stress, such as high irradiation and low water-holding capacity. Surprisingly, this species was not found along the Baltic Sea chronosequence. Nevertheless, it is described as quite common in other sand dunes along the Baltic Coast [91]. In a study in the Curonian Spit, Lithuania, conducted by Jukonienė and Subkaitė [92], *Syntrichia ruralis* var. *ruraliformis* was described as a quite frequent species especially abundant in foredune grasslands. It cannot be ruled out that the species occurred elsewhere in the dunes in the Schaabe spit but not along the investigated transect.

The following later stages of dune succession revealed a shift in community composition along each chronosequence. Pioneer moss species, such as *C. purpureus*, decreased and were supplemented by mosses of later successional stages, such as *Dicranum scoparium* and *Hypnum cupressiforme* var. *lacunosum*. Gypser et al. [35] described *Polytrichum piliferum* as a dominant species of mature biocrusts, which is in line with this study, where *P. piliferum* was only found in the later successional stage close to the forest of the inland dune chronosequence. The mature dune in the Schaabe spit chronosequence was low in moss species richness and was dominated by mosses of the genus *Hypnum*. Comparing the findings of the inland dune in Verden with an official flora mapping in 2015 as part of the habitat’s directive (Council Directive 92/43/EEC) in this area, differences in the moss species composition became obvious. This mapping revealed the presence of 17 moss species in the inland dune area. Only seven species were identified in the present study, three identical to the findings in 2015. That could be because the observation areas and the sampling season might have differed from each other. On the other hand, these differences in the present moss species could be an indicator of a general shift in species composition due to habitat succession or abiotic influences (e.g., drought, rainfall frequency, and intensity).

The dune area close to the forest on the inland dune showed the only growth of lichens along this chronosequence. Only four lichen species of the genus *Cladonia* were found here. Those prefer growing on the sand under acidophil habitat conditions, as in this plot. Furthermore, almost no sand-covering plant material was observed, which could have facilitated the settlement of litter-inhabiting lichen species. Along the dune chronosequence in the Schaabe spit, *Bacidina etayana* was found in the coastal dune habitat. This is typical for this species, which is known to grow exclusively on dune grasses [54]. The intermediate and grey dunes were the lichen species’ richest dune types. Whereas, in the intermediate dune, lichen species primarily grew on plant litter. Lichen species found in these two dune types, such as *Athallia cerinella*, *Lecania cyrtella, Myriolecis hagenii*, *Myriolecis persimilis*, *Physcia tenella*, and *Xanthoria parietina,* do prefer to grow on trees [54]. In the intermediate and grey dunes, tree debris, such as bark or branchlet, was overgrown by these lichen species and provided the favored habitat. As mentioned, in the inland dune, little of such biotic components were found, explaining that none of these species was observed in that dune habitat. In some cases, lichen–litter interactions tend to form a layer independent from biocrusts and further interactions with the underlying sediment. Such formations have to be carefully separated when describing biocrust growth forms. Later successional dune stages were dominated by representatives of the genus *Cladonia*. *Cladonia* primarily inhabits nutrient-poor soils and forests [54]. The *Cladonia* species found grew most likely in the pH range they favor. Hence, in the grey dune area, lichen species preferring moderate acidophilic site conditions (pH 4.9–5.6), such as *C. rei* and *C. foliacea,* were found. While in the mature dune area, lichen species exclusively favoring a strong acidophil sandy substrate were present (e.g., *C. arbuscular* and C. *gracilis*). Comparing the findings of the formerly mentioned flora mapping in 2015 with the present study, three Cladonia species were found in both surveys. Like the mosses, fewer species were identified in the present study probably for the same reasons discussed earlier.

### 4.3. The Effect of Biocrust and Sediment Characteristics, and Nutrient Content on Biocrust Community Composition

Biocrusts are often named ‘ecosystem engineers’ [11,76] for their multiple ecological functions, such as their contribution to soil stabilization and fertilization. Primary production, element solubilization and mineralization, and nitrogen fixation [9,23,93] are processes mediated by microbial activities within biocrusts. A general increase in the nutrient concentration within biocrusts along both transects could be observed, which is in line with previous studies [28,43,94]. These findings indicate that nutrients accumulate within biocrusts and the different functional groups of biocrusts take different weighted parts in this process. In the present study, nutrient contents (C_t_, N_t_, and P_t_) were correlated with the species richness of all investigated phototrophic organism groups (algae, mosses, and lichens) to distinguish between those parts. Although no significant correlation between the overall phototrophic species richness and the different nutrient contents could be observed, more stable biocrusts dominating later dune successional stages are associated with an increase in the total C, N, and P contents along each chronosequence. Considering the three investigated phototrophic groups separately, the impact of the moss community on nutrient enrichment becomes obvious. Due to the higher accumulation of biomass by this phototrophic group, coherence becomes obvious. The results indicate that species composition rather than the overall species richness shapes the biocrust function. Mosses accumulate organic matter and nutrients within their biomass and biocrust-adherent sediment layers. Due to their rhizoids, they are able to penetrate deeper into the sediment compared to lichens and algae. They distribute organic matter and nutrients more spaciously than it is possible for thinner algae-dominated biocrusts [95]. Concerning the moss species richness, a link to the phosphorus cycle can be considered. Phosphorus is an essential element for the growth of all phototropic organisms. The bioavailability of this element in dunes is low due to its strong binding to unweathered minerals [26]. Schulz et al. [43] could reveal phosphorus as a driver for the establishment and cyanobacterial/algal species composition. The later moss community might benefit from the previously established algal biocrust community and binds further available amounts of phosphorus within their biomass. Moreover, the potential for biocrusts to be carbon sinks was investigated in recent studies [96,97]. These results point to another forward-looking ecological function of biocrusts. Their potential influence in reducing atmospheric carbon by inoculation within their biomass highlights their importance in ecosystem conservation and climate change.

## 5. Conclusions

The study results pointed to a conspicuous shift of the phototrophic biocrust community with the development of the dunes along each chronosequence, as reflected in different successional stages. Furthermore, the different geographical regions revealed a considerable impact on the biocrust species composition, which can be explained by habitat-specific factors. The enrichment of organic matter and nutrient contents along with increased soil moisture and stability are the key functions that originate from the metabolic activities of the different stages of biocrusts. Consequently, biocrusts strongly shape dune development and contribute to geomorphological processes. In addition, the biocrust key functions offer opportunities for rehabilitation and restoration approaches, such as preventing sediment erosion in coastal dune areas.

## Figures and Tables

**Table 1:** Overview of all measured biocrust and sediment characteristics (uppermost sediment layer *, as well as biocrust) ^1^.

Study Site	Dune Area	Sampling Plot	Water Content	OM	pH
			(% FW)	(% DW)	(CaCO_3_)
Schaabe (Rügen)	Coastal dune	FD	0.52 ± 0.83 *	0.11 ± 0.01 *	6.39 *
		ID	0.16 ± 0.05	1.38 ± 0.3	6.13 *
		GD	0.39 ± 0.04	7.14 ± 0.89	5.58 *
		MD	1.22 ± 0.56	25.19 ± 7.7	3.98 *
Verden (Aller)	Inland dune	DC	0.02 ± 0.01 *	<0.1 *	4.49 *
		DS	0.16 ± 0.04	3.3 ± 0.72	3.68 *
		DF	1.73 ± 0.05	24.03 ± 2.66	3.35 *

^1^ Water content (in % of fresh weight) and organic matter content (OM, in % of dry weight, after combustion of dry samples at 450 °C) are given in percent. Parameters were measured in triplicates. The pH was only measured once per sampling plot. All data represent mean values ± standard deviation. Site: Schaabe. Dune types: FD = foredune, ID = intermediate dune, GD = grey dune, and MD = mature dune. Site: Verden. Sampling plots: DC = dune center, DS = dune slope, and DF = dune forest.

**Table 2:** Overview of all measured nutrient contents of the uppermost sediment layer * (if no biocrusts were visible), as well as biocrusts ^2^.

Study Site	Dune Area	Sampling Plot	C_t_ (g kg^−1^ DW)	N_t_ (g kg^−1^ DW)	P_t_ (mg kg ^−1^ DW)
Schaabe (Rügen)	Coastal dune	FD	0.57 ± 0.09 *	0.1 ± 0 *	113.90 ± 12.61 *
		ID	6.63 ± 0.68	0.42 ± 0.11	148.92 ± 22.85
		GD	35.54 ± 10.39	1.3 ± 0.1	173.16 ± 16.34
		MD	84.32 ± 40.69	3.3 ± 1.51	172.23 ± 69.04
Verden (Aller)	Inland dune	DC	1.16 ± 0.36 *	0.2 ± 0 *	29.89 ± 2.29 *
		DS	15.34 ± 3.02	0.81 ± 0.25	71.85 ± 11.44
		DF	99.63 ± 4.59	4.72 ± 0.24	291.25 ± 58.11

^2^ Total phosphate (Pt) concentration is expressed as mg kg^−1^ dry weight. Total carbon (Ct) and nitrogen (Nt) concentrations are expressed as g kg^−1^ dry weight. Parameters were measured in triplicates. All data represent mean values ± standard deviation. Site: Schaabe. Dune types: FD = foredune, ID = intermediate dune, GD = grey dune, and MD = mature dune. Site: Verden. Sampling plots: DC = dune center, DS = dune slope, and DF = dune forest.

**Table 3:** Green algal and cyanobacterial species in biocrusts were sampled at six different dune plots ^3^.

Phylum, Class	Species	Inland Dune			Coastal Dune		
		DC	DS	DF	ID	GD	MD
**Cyanobacteria**							
Cyanophyceae	*Nostoc* cf. *edaphicum*						
**Chlorophyta**							
Chlorophyceae	*Bracteacoccus* sp.						
	*Chlorolobion* sp.						
	*Coelastrella* sp.						
Trebouxiophyceae	*Chloroidium ellipsoideum*						
	*Chloroidium* sp.						
	*Diplosphaera chodatii*						
	*Elliptochloris subsphaerica*						
	*Myrmecia* cf. *irregularis*						
	*Parietochloris alveolaris*						
	*Stichococcus allas*						
	*Stichococcus* cf. *bacillaris*						
	*Watanabea* cf. *acidophila*						
**Charophyta**							
Klebsormidiophyceae	*Klebsormidium* cf. *subtile*						

^3^ Site: coastal dune (Schaabe). Dune types: ID = intermediate dune, GD = grey dune, and MD = mature dune. Site: inland dune (Verden). Sampling plots: DC = dune center, DS = dune slope, and DF = dune forest. Dark grey shading indicates the occurrence of the species at that site. Light shading parts indicate no detection of the species at the respective location. Bold font in the first table column is used to highlight the algal phyla under which the corresponding classes are listed.

**Table 4:** Moss species in biocrusts were sampled at five different dune plots ^4^.

Phylum, Class	Species	Inland Dune		Coastal Dune		
		DS	DF	ID	GD	MD
**Bryophyta**						
Bryopsida	*Brachythecium albicans*					
	*Bryum capillare*					
	*Campylopus introflexus*					
	*Ceratodon purpureus*					
	*Dicranum scoparium*					
	*Hypnum cupressiforme*					
	*Hypnum cupressiforme* var. *lacunosum*					
	*Hypnum jutlandicum*					
	*Pohlia nutans*					
	*Ptychostomum capillare*					
	*Ptychostomum imbrictulum*					
	*Racomitrium elongatum*					
	*Syntrichia ruraliformis*					
Polytrichopsida	*Polytrichum piliferum*					
**Marchantiophyta**						
Jungermanniopsida	*Cephaloziella divaricata*					
	*Ptilidium ciliatum*					

^4^ Site: coastal dune (Schaabe). Dune types: ID = intermediate dune, GD = grey dune, and MD = mature dune. Site: inland dune (Verden). Sampling plots: DS = dune slope and DF = dune forest. Dark grey shading indicates the occurrence of the species at that site. Light shading parts indicate no detection of the species at the respective location. Bold font in the first table column is used to highlight the moss phyla under which the corresponding classes are listed.

**Table 5:** Lichen species in biocrusts were sampled at four different dune plots and are listed by the order of lichen-forming fungi (Mycobiont) ^5^.

Phylum, Class, and Order (Mycobiont)		Species	Inland Dune	Coastal Dune		
			DF	ID	GD	MD
**Ascomycota**						
*Lecanoromycetes*						
Baeomycetales	*Placynthiella uliginosa*					
Lecanorales	*Bacidina etayana*					
	*Cladonia arbuscula*					
	*Cladonia chlorophaea*					
	*Cladonia conista*					
	*Cladonia fimbriata*					
	*Cladonia foliacea*					
	*Cladonia furcata*					
	*Cladonia gracilis*					
	*Cladonia humilis*					
	*Cladonia phyllophora*					
	*Cladonia portentosa*					
	*Cladonia ramulosa*					
	*Cladonia rei*					
	*Cladonia scabriuscula*					
	*Cladonia uncialis* ssp. *biuncialis*					
	*Evernia prunastri*					
	*Hypogymnia physodes*					
	*Lecania cyrtella*					
	*Myriolecis hagenii*					
	*Myriolecis persimilis*					
	*Micarea misella*					
	*Parmelia sulcata*					
Peltigerales	*Peltigera extenuata*					
Teloschistales	*Athallia cerinella*					
	*Physcia tenella*					
	*Xanthoria parietina*					

^5^ Site: coastal dune (Schaabe). Dune types: ID = intermediate dune, GD = grey dune, and MD = mature dune. Site: inland dune (Verden). Sampling plot: DF = dune forest. Dark grey shading indicates the occurrence of the species at that site. Light shading parts indicate no detection of the species at the respective location. Bold font in the first table column is used to highlight the detected lichen phylum. Italic font in the first table column is used to highlight the lichen class under which the corresponding orders (Mycobiont) are listed.

## Data Availability

Environmental data will be available in the Pangaea database. The accession number for the environmental parameters is https://doi.pangaea.de/10.1594/PANGAEA.947837 (accessed on 23 March 2022). For the taxonomic data, see https://doi.pangaea.de/10.1594/PANGAEA.947829 (accessed on 23 March 2022).

## Supplementary Materials

The following supporting information can be downloaded at https://www.mdpi.com/article/10.3390/biology12010058/s1, Table S1: Overview of all defined functional biotic and abiotic groups used to determine the dune surface coverage. Abbreviations of these groups, a short description, a color code, and visual examples of each functional group are given. Table S2: Chlorophyll *a* content (mg m^−2^) for the biocrust or sediment (*) along both dune chronosequences. Table S3: Lichen species in the biocrusts are listed by the order of lichen-forming fungi (Mycobiont). Columns differentiate between lichens’ occurrence and characterize the preliminary settled pH range. Abbreviations: e = extreme, v = very, q = quite, and m = moderate. Figure S1: A sampling plot (1 m^2^) divided into 16 equal subplots (0.0625 m^2^) with a 25 cm × 25 cm (0.0625 m^2^) grid of 25 intersections. A metal pin was dropped next to each intersection and the ground covering the functional group, including bare sediment, was recorded. Figure S2: Venn diagrams showing the total species number of (A) algae, (B) mosses, and (C) lichens at the two sampling sites. The number of species that were found at both sites is represented as an overlap between the two circles. Figure S3: Table showing Pearson correlation coefficients to reveal correlations between nutrient contents, which might affect or be affected by the richness of the phototrophic biocrust community. A plot of moss richness in biocrusts over the total phosphorus content in the biocrusts from both sites. The line indicates the best linear fit (*p* = 0.115 (ANOVA)).
