# Supplementary material for: Successional Development of the Phototrophic Community in Biological Soil Crusts on Coastal and Inland Dunes
Source: Biology (Basel). 2022 Dec 29;12(1):58. doi: 10.3390/biology12010058 (PMC9856012; doi:10.3390/biology12010058)

Successional development of the phototrophic community in biological soil crusts on coastal and inland dunes

Sandra Kammann[1], Ulf Schiefelbein[2], Christian Dolnik[3], Tatiana Mikhailyuk[4], Eduard Demchenko[4], Ulf Karsten[1], Karin Glaser[1]

[1] Institute for Biological Sciences, Applied Ecology and Phycology, Rostock University, Albert-Einstein-Straße 3, 18059 Rostock, Germany

[2] Botanical Garden, University of Rostock, Schwaansche Straße 2, 18055 Rostock, Germany

[3] Institute for Natural Resource Conservation, Landscape Ecology, Kiel University, Ohlshausenstr. 40, 24098 Kiel, Germany

[4] M. G. Kholodny Institute of Botany, National Academy of Science of Ukraine, Tereschenkivska St. 2, Kyiv UA-01601, Ukraine

Table S1: Overview of all defined functional biotic and abiotic groups used to determine the dune surface coverage. Abbreviations of these groups, a short description, a color code, and visual examples of each functional group are given.

Category	Short	Description	Code	
Vascular plants	VP	Tracheophyta		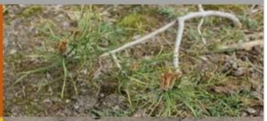
Bare sediment/ sand	Ba	No vegetation or biocrust visible, loose sand layer		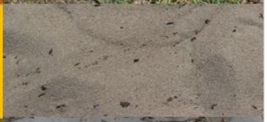
Litter	Li	Litter layer i.e. needles, leaves, branches		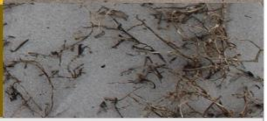
**Biocrust**				
Green algae	GA	Early successional stage, slightly brittle, <3 mm thick, greenish-shimmering		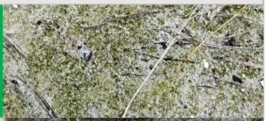
Green algae-dominated /mosses	GA/M	Stable green crust, occasionally mosses		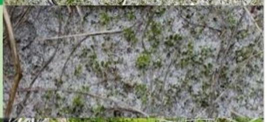
Moss-dominated	MD	biocrust dominated by mosses, partly many algae species (mostly green algae), several cm thick/high		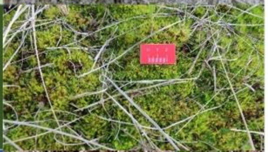
Moss-dominated /lichens	MD/L	MD-type, occasionally lichens (mostly chlorolichens), many algae species (mostly green algae), several cm thick/high		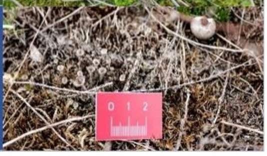

Figure S1: Sampling plot (1 m^2) divided into 16 equal subplots (0.0625 m^2) with 25 cm x 25 cm (0.0625 m^2) grid of 25 intersections. A metal pin was dropped next to each intersection, and the ground covering functional group, including bare sediment, was recorded.

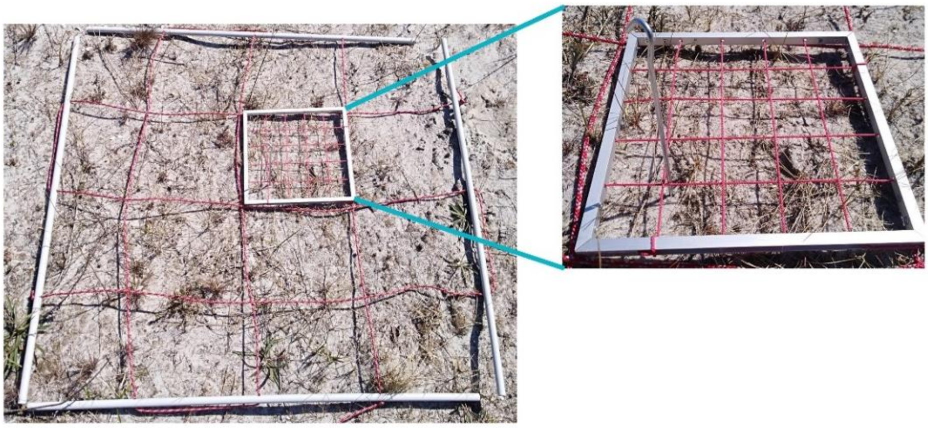

Table S2: Chlorophyll *a* content (mg m^{-2}) for biocrusts or sediment (*) along both dune chronosequences.

Study site	Dune area	Sampling plot	Chl *a* (mg m^{-2})
Schaabe (Rügen)	Coastal dune	FD	0*
		ID	161.34 ± 56.51
		GD	150.92 ± 26.49
		MD	287.63 ± 49.07
Verden (Aller)	Inland dune	DC	5.86*
		DS	112.07 ± 20.0
		DF	210.80 ± 109.01

Figure S2: Venn diagrams showing the total species number of **(A)** algae, **(B)** mosses, and **(C)** lichens at the two sampling sites. The number of species that were found at both sites are represented as an overlap between the two circles.

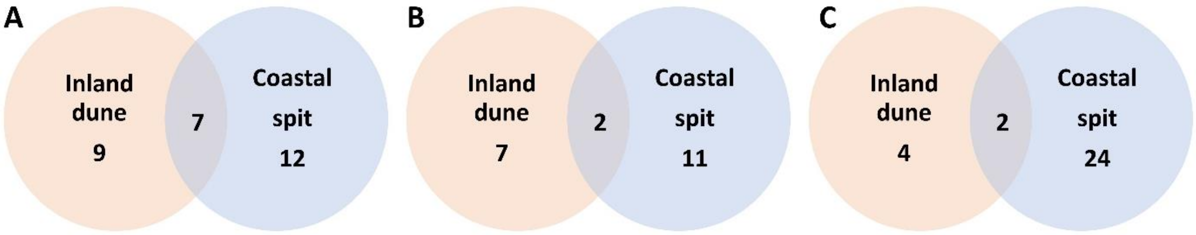

Table S3: Lichen species in biocrusts. Listed by the order of lichen-forming fungi (Mycobiont). Columns differentiating between lichens occurrence and characterizing the preliminary settled pH range; Abbreviations: e=extreme, v=very, q=quite, m=moderate.

**Phylum**, class, order (Mycobiont)	Species	Occur on	pH range
**Ascomycota**			
Lecanoromycetes			
Baeomycetales	*Placynthiella uliginosa*	Sand	e.acidoph.-q.acidoph.
Lecanorales	*Bacidina etayana*	Litter	
	Cladonia arbuscula	Sand	e.acidoph.-m.acidoph.-(subneutroph)
	Cladonia chlorophaea	Sand	(e.acidoph.-)v.acidoph.-m.acidoph.
	Cladonia conista	Sand	acidoph.
	Cladonia fimbriata	Sand	q.acidoph.-m.acidoph.-(subneutroph)
	Cladonia foliacea	Sand	(q.acidoph.-)m.acidoph.
	Cladonia furcata	Sand	q.acidoph.-subneutroph
	Cladonia gracilis	Sand	e.acidoph.-m.acidoph.
	Cladonia humilis	Sand	q.acidoph.-subneutroph
	Cladonia phyllophora	Sand	q.acidoph.-m.acidoph.
	Cladonia portentosa	Sand	e.acidoph.-m.acidoph.-(subneutroph)
	Cladonia ramulosa	Sand	v.acidoph.-q.acidoph.
	Cladonia rei	Sand	m.acidoph.-subneutroph
	Cladonia scabriuscula	Sand	m.acidoph.-subneutroph
	Cladonia uncialis ssp. *biuncialis*	Sand	e.acidoph.-m.acidoph.-(subneutroph)
	Evernia prunastri	Sand	v.acidoph.-m.acidoph.
	Hypogymnia physodes	Sand	v.acidoph.-m.acidoph.
	Lecania cyrtella	Litter	subneutroph
	Myriolecis hagenii	Litter	subneutroph.-basiph.
	Myriolecis persimilis	Litter	subneutroph.-neutroph.
	Micarea misella	Litter	v.acidoph.-q.acidoph.
	Parmelia sulcata	Sand	(q.acidoph.-)m.acidoph.
Peltigerales	*Peltigera extenuata*	Sand	q.acidoph.-subneutroph.
Teloschistales	*Athallia cerinella*	Litter	subneutoph.
	Physcia tenella	Litter	subneutoph.-m.basiph.
	Xanthoria parietina	Litter	subneutoph.m.basiph.

Figure S3: Table showing Pearson correlation coefficients to reveal correlations between nutrient contents, which might affect or be affected by the richness of the phototrophic biocrust community. Plot of moss richness in biocrusts over the total phosphorus content in the biocrusts from both sites. The line indicates the best linear fit ($p = 0.115$ (ANOVA)).

	Richness All	Richness Algae	Richness Mosses	Richness Lichens
Ct (g/kg)	0.196	0.021	0.432	0.118
Nt (g/kg)	0.154	0.016	0.408	0.059
Pt (g/kg)	0.441	0.102	0.649	0.396

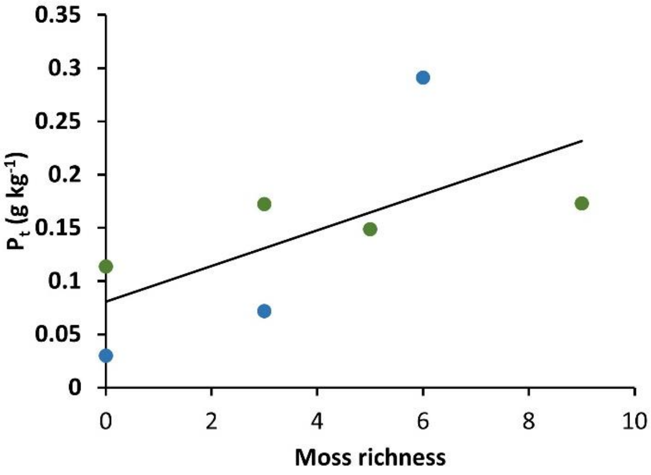

Supplement: Supplementary file 1 [file biology-12-00058-s001.zip › biology-1995647-supplementary.pdf]
